# Observation on the Efficacy of Hyperbaric Oxygen as Adjuvant Therapy for Breast Cancer‐Related Wounds: A Case Report

**DOI:** 10.1002/cnr2.70563

**Published:** 2026-04-29

**Authors:** Fengrong Tang, Lei Zhang, Shufang Lu, Xiaochen Mo, Xiaoxiao Duan

**Affiliations:** ^1^ Nursing Department, Minhang Branch Fudan University Shanghai Cancer Center Shanghai China

**Keywords:** breast cancer, exudate, hyperbaric oxygen, malignant wounds, nursing, odor

## Abstract

**Background:**

Hyperbaric oxygen therapy (HBOT) is widely used as a therapeutic intervention for various types of wounds. Studies have shown that in addition to improving local oxygen supply to the wound site, HBOT can also influence cytokine activity, promote the survival of tissue mesh, and thereby facilitate wound healing. Moreover, HBOT inhibits the growth and reproduction of various pathogenic bacteria and enhances the phagocytic capacity of leukocytes, effectively preventing and controlling infection.

**Case:**

This case study involves a 61‐year‐old female diagnosed with invasive micropapillary carcinoma, who underwent a combination of treatments including radiotherapy, chemotherapy, immunotherapy, and targeted therapy. Nine years post‐surgery, the patient experienced a recurrence of left breast cancer, accompanied by cancer wounds with exudate, edema, and ulceration in multiple areas such as the chest and back. Following comprehensive treatment consisting of anti‐infective therapy, nutritional support, and edema management, along with three courses of adjuvant HBOT, the patient showed significant improvement in wound exudate and odor, resolution of lymphedema, and a reduction in wound volume, although the wound area did not show notable change.

**Conclusion:**

This study implemented a comprehensive care plan for patients with malignant wounds, including measures such as wound debridement, anti‐infection treatment, nutritional support, and HBOT. Following the integrated intervention, symptoms including wound exudate, odor, and lymphedema were alleviated, and wound volume was reduced, although no significant change was observed in wound area. The primary benefit of HBOT in malignant wound management lies in symptom control, while its effect on structural wound healing appears limited. Its specific role within multidisciplinary comprehensive care requires further investigation.

## 1 Introduction

Breast cancer remains one of the most common cancers worldwide [1]. In 2024, both the incidence and mortality rates of breast cancer in China have continued to rise. Despite advancements in early screening and treatment, it persists as a leading cause of cancer‐related death among women in China [2]. Globally, 2022 saw 2.3 million new breast cancer cases and 670 000 deaths. It is the most frequently diagnosed cancer in women, accounting for 25% of all cancer cases and 15.5% of cancer‐related deaths [3].

The incidence of malignant fungating wounds (MFWs) ranges from 5% to 10%, with 62% of these occurring in breast cancer patients [4, 5]. MFWs present with symptoms including heavy bleeding, severe pain, foul odor, copious exudate, and damage to the surrounding skin.

Hyperbaric oxygen therapy (HBOT) involves breathing 100% oxygen in a pressurized environment exceeding 0.1 MPa (1.0 ata) to treat various medical conditions [6]. HBOT exerts antibacterial effects against anaerobic organisms and modulates immune function. By significantly increasing oxygen partial pressure at the infection site, it effectively eliminates anaerobic bacteria [7]. The vasoconstrictive effect of HBOT can alleviate poor tissue perfusion and impaired lymphatic return caused by limb swelling. The concomitant correction of tissue hypoxia further reduces edema, although its specific indications and mechanisms of action remain subjects of debate [8].

Tumor hypoxia is a key characteristic of malignant wounds. HBOT counteracts this by increasing dissolved oxygen content in the plasma, thereby enhancing oxygen delivery to tissues. This process mitigates tumor hypoxia and reduces cancer cells' reliance on anaerobic glycolysis, ultimately altering their metabolic state [9, 10]. This case report describes the adjunctive use of HBOT for a malignant wound secondary to breast cancer. After three treatment cycles, the patient exhibited significant improvement in wound exudate, odor, and lymphedema.

## 2 Case Presentation

A 61‐year‐old female patient (height: 160 cm; weight: 51 kg; BMI: 19.9 kg/m^2^) was admitted to the Fudan University Shanghai Cancer Center, Minhang Branch, presenting with recurrent left breast cancer accompanied by fluid accumulation (effusion), edema, and ulceration in the chest and back. The patient reported chest tightness and shortness of breath but was afebrile. There was no history of drug allergies, infectious diseases, or significant family medical history.

The patient had undergone a modified radical mastectomy for left breast cancer in 2015, with a pathological diagnosis of invasive micropapillary carcinoma. Postoperative management included a combination of radiotherapy, chemotherapy, immunotherapy, and targeted therapy. Upon admission, her vital signs were stable: body temperature, 36.5°C; pulse, 86 beats/min; respiratory rate, 20 breaths/min; and blood pressure, 115/65 mmHg.

Upon admission, routine laboratory tests revealed the following: red blood cell count 2.44 × 10^12^/L (↓), hemoglobin 89 g/L (↓), albumin 30 g/L (↓), cardiac troponin T (cTnT) 0.063 ng/mL (↑), d‐dimer 5.71 mg/L (↑), and white blood cell count 26.30 × 10^9/L (↑). A non‐contrast chest CT scan showed scattered fibrotic foci in both lungs, atelectasis in the lower lobes, and bilateral pleural effusion.

The primary treatments administered during this hospitalization included supportive care with antibiotics, nutritional support, edema reduction, HBOT, and wound dressing changes. The patient was discharged on May 6, 2025. At discharge, the laboratory results were as follows: red blood cell count 2.70 × 10^12^/L (decreased), hemoglobin 92 g/L (decreased), albumin 31 g/L (decreased), d‐dimer 1.91 mg/L (elevated), and white blood cell count 4.26 × 10^9^/L.

### 2.1 The Wound Measurement, Assessment, and Dressing Change Procedures Were Conducted as Follows

All wound management and assessments in this study were independently conducted by two nurses certified in wound care to ensure consistency in procedures and observations.

#### 2.1.1 Wound Measurement

Wound dimensions were measured using a sterile ruler. The length was recorded along the longest axis of the wound, and the width was measured perpendicular to the length at the widest point. Depth was determined by measuring the vertical distance from the wound surface to its deepest point using a sterile probe. Wound volume was calculated using the ellipsoid formula: Volume = Length × Width × Depth × 0.52.

#### 2.1.2 Clinical Assessment

At each dressing change, the two nurses independently assessed the wound using standardized tools. Exudate volume was graded according to the TELER Malignant Wound Assessment Tool, and odor was assessed using the Grocott Scale. All assessments were performed strictly in accordance with the definitions provided in each scale. After independent scoring, the two nurses compared and verified their results to ensure reliability.

#### 2.1.3 The Method of Dressing Change

First, wound cleansing and debridement: Following the principles of surgical aseptic technique, the wound was initially cleansed with normal saline. Necrotic tissue and slough surrounding the wound were excised using sterile scissors. The wound bed was then wiped with 0.9% sodium chloride cotton balls to remove exudate, followed by turbulent irrigation with 20 mL of 0.9% sodium saline using a syringe [11].

Second, medicated moist dressing and change: After cleansing, the wound was air‐dried. Due to the presence of foul odor and significant exudate, a moist dressing with chymotrypsin was applied to control the odor. Finally, the wound was covered with a silver‐ion dressing and secured with a gauze bandage.

Concurrently, a bacterial culture and antibiotic susceptibility test of the wound were performed. Based on the results, appropriate sensitive antibiotics were selected. When necessary, effective systemic antibiotic therapy was administered to prevent hematogenous spread of the wound infection [12].

### 2.2 HBOT and Follow‐Up

HBOT enhances chemosensitivity by improving oxygen supply to hypoxic tissue regions, while also reducing toxicity and exerting a sensitizing effect. Additionally. It is also effective in preventing and managing late adverse effects of radiotherapy. HBOT can reduce the risk of pulmonary arteritis, prevent damage to pulmonary capillaries or mitigate the extent of such damage, alleviate pulmonary edema, improve pulmonary ventilation, and decrease the incidence of pulmonary infections [13].

The treatment protocol, initiated on March 20, 2025, was administered as follows: oxygen was delivered via a mask with minimal‐resistance nebulization at a pressure of 2.0 ata (1 ata = 760 mmHg). Each session consisted of 20 min of compression, 60 min of stable oxygen inhalation, and 30 min of decompression, conducted once daily. A full course comprised 10 sessions, and the patient received three courses in total.

**FIGURE 1:**
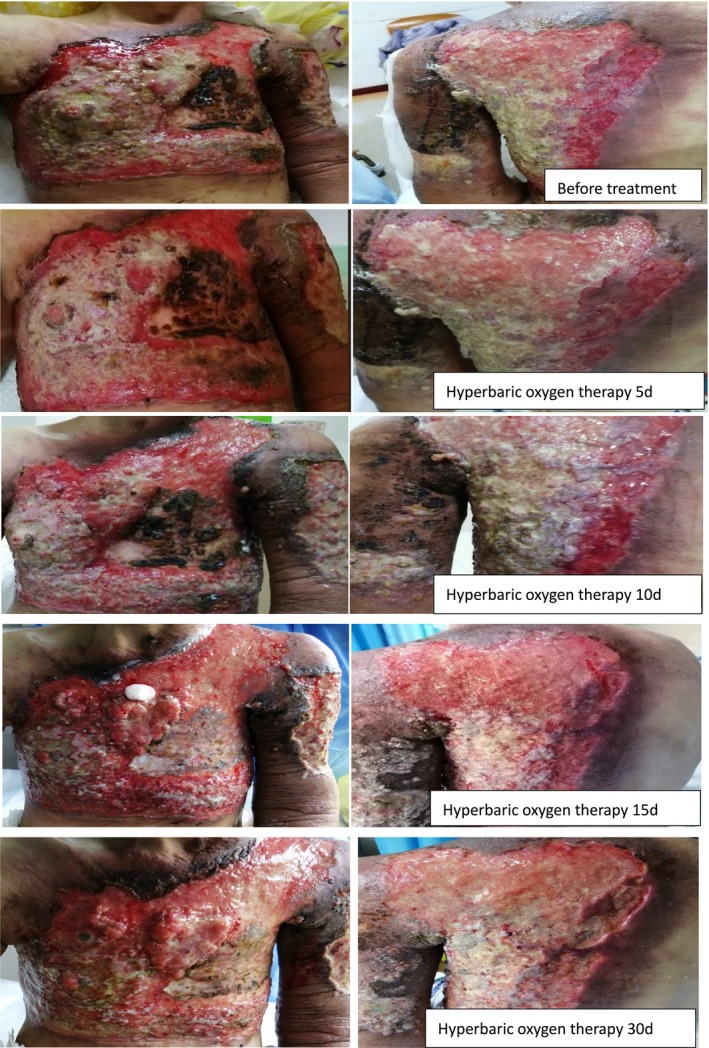
Hyperbaric oxygen‐assisted treatment of wounds for 30 days.

Wound recovery was assessed before treatment and at 5, 10, 15, and 30 days after the initiation of HBOT. After 30 days of adjunctive HBOT, the patient showed reduced wound exudate and odor, along with resolved lymphedema. Although the surface area of the malignant wound remained unchanged, its height above the skin decreased slightly. The volume of the protruding malignant wound reduced from 152 cm^3^ before treatment to 101 cm^3^ on day 15 of HBOT, indicating a decrease in wound volume. For details, refer to Table 1 and Figure 1.

**TABLE 1:** Upper limb lymphedema and wound exudate and odor assessment after 30 days of hyperbaric oxygen therapy.

Hyperbaric oxygen therapy days	Upper limb lymphedema								Wound odor level	Wound exudate	Wound area (cm^2^)	Wound volume (cm^3^)
	place	Tiger's mouth	Wrist striation	On the horizontal lines on the wrist 10 cm	Under the elbow 10 cm	Elbow	On the elbow 10 cm	Armpit level				
When not treated	Right upper limb	21	21	21.2	26	28	27.5	26	Level 1	Level 0	1030	152
	Left upper limb	21	21.5	21.5	23.6	33						
5 days	Right upper limb	20.5	18.5	19.3	25	26.3	27.5	26	Level 2	Level 1	1030	153
	Left upper limb	21.4	19.9	21.5	22.6	33						
10 days	Right upper limb	19	18.9	21.9	24.7	28.7	28	28	Level 3	Level 2	1030	152
	Left upper limb	21.1	19.9	22.1	23.1	33.2						
15 days	Right upper limb	18.3	17.5	20.4	24	29.9	28.5	28	Level 3	Level 3	1030	101
	Left upper limb	20.1	19.1	21.2	23.6	30						
30 days	Right upper limb	17.9	16.2	19.5	23.4	27.2	24.8	25.2	Level 4	Level 3	1030	101
	Left upper limb	20.7	18	20.7	22.6	31.6						

*Wound exudate leakage* [14]: was assessed using the 2001 TELER malignant tumor wound assessment tool. The exudate leakage index classifies leakage on a scale from 0 to 5, where a higher score indicates less leakage. The grading is as follows:

Grade 0: Dressing and bed clothes are soaked.

Grade 1: Dressing and bed clothes are damp.

Grade 2: Dressing is soaked, and bed clothes are wet.

Grade 3: Dressing is soaked, and bed clothes are contaminated with an area the size of a coin.

Grade 4: Only the dressing is soaked.

Grade 5: Only the dressing is contaminated (Table 1).

*Wound odor* [15]: was evaluated using the Grocott Wound Odor Assessment Scale, which grades odor from 0 to 5. A higher grade indicates a lighter odor (Table 1).

*Follow‐up*: The patient was followed up on June 6, 2025. Routine laboratory tests revealed the following: red blood cell count 2.75 × 10^12^/L (decreased), hemoglobin 99 g/L (decreased), albumin 33 g/L (decreased), cTnT 0.048 ng/mL (elevated), d‐dimer 1.63 mg/L (elevated), and white blood cell count 5.16 × 10^9^/L. A non‐contrast chest CT scan showed significant resolution of bilateral pleural effusion and near‐complete re‐expansion of atelectasis in both lower lobes. The patient's laboratory parameters had improved, and clinical symptoms had alleviated.

Wound assessment indicated that post‐discharge, wound exudate and odor were significantly reduced compared to pre‐discharge levels, and lymphedema was effectively controlled, having subsided without further progression. However, no significant change was observed in wound area or volume compared to discharge, and substantial structural healing was not evident. The patient was transferred to another hospital for skin grafting on June 15 and subsequently passed away due to infection on July 2.

## 3 Discussion

The patient in this case received multimodal comprehensive treatment, including anti‐infection therapy, nutritional support, wound debridement, antimicrobial dressings, complex lymphedema therapy, and HBOT. The observed improvements in laboratory parameters, imaging findings, and clinical symptoms resulted from the combined effects of these multiple interventions and cannot be attributed to HBOT alone. The following discussion aims to analyze the potential mechanisms underlying the changes in various indicators and to explore the possible synergistic role of HBOT within the comprehensive treatment plan, rather than its independent therapeutic effect.

### 3.1 Dynamic Changes in Laboratory and Imaging Findings and the Potential Role of HBOT

Significant dynamic changes in laboratory and imaging findings before and after treatment objectively reflected systemic improvement, which corresponded closely with the alleviation of clinical symptoms. As part of the comprehensive intervention, HBOT may have contributed to this improvement through multiple mechanisms.

Initial laboratory tests upon admission revealed multi‐system dysfunction: ① Severe infection: A white blood cell count of 26.30 × 10^9^/L indicated a systemic inflammatory response triggered by local wound infection. ② Nutritional depletion: Hemoglobin was 89 g/L (moderate anemia) and albumin was 30 g/L (hypoalbuminemia), reflecting a catabolic state. The hypoalbuminemia directly contributed to the subsequent imaging finding of pleural effusion. ③ Hypercoagulable state: A d‐dimer level of 5.71 mg/L suggested potential impairment of microcirculatory perfusion in the wound. ④ Myocardial injury: A cTnT level of 0.063 ng/mL indicated subclinical myocardial injury due to infection and anemia, providing an objective basis for the patient's chest tightness and shortness of breath. ⑤ Chest CT scan showed scattered fibrotic foci in both lungs, atelectasis in the lower lobes, and bilateral pleural effusion. The bilateral pleural effusion was directly related to hypoalbuminemia, where decreased plasma colloid osmotic pressure led to fluid extravasation into the pleural space, forming a transudative effusion. Compression of lung tissue by the effusion resulted in atelectasis of the lower lobes, which constituted a key pathological basis for the patient's symptoms.

At follow‐up (June 6), laboratory parameters showed comprehensive improvement: white blood cell count normalized, hemoglobin increased to 99 g/L, albumin rose to 33 g/L, d‐dimer decreased to 1.63 mg/L, and cTnT declined to 0.048 ng/mL. Concurrent CT imaging revealed significant resolution of bilateral pleural effusion and near‐complete re‐expansion of the lower lobes. This improvement was directly linked to the rise in albumin levels—as nutritional status improved and plasma colloid osmotic pressure recovered, the effusion was absorbed and lung tissue re‐expanded.

HBOT may have played a synergistic role in this improvement process [16, 17, 18, 19]: ① Infection control: HBOT enhances neutrophil phagocytic function and bactericidal capacity while inhibiting anaerobic bacterial growth. ② Support for tissue repair: By improving tissue oxygenation, HBOT may promote protein synthesis and fibroblast proliferation, thereby enhancing the efficiency of nutrient utilization. ③ Alleviation of hypercoagulability: HBOT reduces blood viscosity, improves endothelial function, and decreases platelet aggregation. ④ Promotion of edema resolution: HBOT improves microcirculatory perfusion, accelerating the absorption of pleural effusion and tissue edema. It is important to emphasize, however, that measures such as anti‐infective therapy and nutritional support themselves are the primary drivers of improvement in the aforementioned indicators, making it impossible to quantify the effect of HBOT in isolation.

### 3.2 The Role of HBOT in Controlling Symptoms of Malignant Wounds

Malignant wounds are a common complication in patients with advanced breast cancer, often accompanied by foul odor, significant exudate, and surrounding lymphedema, which severely impair the patient's quality of life [12]. The results of this study show that, in addition to a comprehensive care plan including wound debridement, antimicrobial dressings, and nutritional support, the combination with HBOT led to some alleviation of wound exudate, odor, and upper limb lymphedema. However, no significant reduction in wound surface area or volume was observed.

The findings indicate that in this case, the primary benefit of HBOT was oriented more toward symptom control rather than structural wound healing. HBOT may reduce infection‐related odor and exudate by increasing tissue oxygen partial pressure, improving local ischemic and hypoxic conditions, and inhibiting the growth of anaerobic bacteria and certain fungi [20]. Meanwhile, the vasoconstrictive effect of HBOT could help alleviate tissue edema, while the hyperoxic environment may enhance the functions of fibroblasts and macrophages, thereby promoting the clearance of necrotic tissue. However, the cancerous wound itself consists of invasively growing tumor tissue, and the repair of its structural defect is limited by the ongoing progression of the tumor—this may reasonably explain the lack of significant improvement in wound area observed in this case.

Friedman et al. [21, 22] and Flegg et al. [23, 24] suggested through mathematical models in previous studies that intermittent HBOT may help improve the healing environment of chronic ischemic wounds. It should be noted, however, that these studies are primarily based on non‐malignant chronic wound models. The pathophysiological mechanisms of malignant wounds differ fundamentally from those of non‐malignant wounds, as the former involve continuous tumor cell proliferation and tissue destruction. Therefore, the mechanism of HBOT in malignant wounds is more complex, primarily manifesting in symptom control (odor, exudate, edema) with limited effects on the structural healing of malignant wounds.

### 3.3 Effect of HBOT on Alleviating Breast Cancer‐Related Lymphedema (BCRL)

BCRL is characterized by the accumulation of protein‐rich fluid in the interstitial spaces due to impaired lymphatic drainage, clinically presenting as heaviness, swelling, and limited mobility of the upper limb [25]. In this study, we observed that after 1 month of adjunctive HBOT, patients exhibited a significant reduction in upper limb lymphedema compared to the pretreatment condition. The potential mechanisms by which HBOT alleviates lymphedema may include [26]: (1) vasoconstriction induced by the hyperoxic environment, which may reduce capillary leakage; (2) increased oxygen partial pressure promoting lymphangiogenesis and enhancing lymphatic return; and (3) mitigation of local inflammatory responses and improvement of microcirculatory perfusion. These findings are consistent with previous reports, such as that by Gong et al. [27], suggesting that HBOT may hold clinical value in alleviating symptoms associated with BCRL. However, it should be noted that the reduction in lymphedema observed in this study resulted from a comprehensive intervention, making it difficult to attribute the improvement solely to HBOT alone. Furthermore, the reduction in lymphedema was correlated with a decrease in wound volume. Lymphedema leads to localized fluid accumulation, causing swelling around the wound and in the affected limb, thereby increasing the overall wound volume. As edema is controlled and swelling subsides, wound volume decreases accordingly, although the wound area may remain unchanged due to scar formation or tissue deficit. Therefore, the reduction in volume primarily reflects tissue contraction resulting from edema resolution, rather than structural wound healing.

## 4 Conclusion

This case demonstrates that the integration of HBOT into a multimodal comprehensive treatment regimen may contribute, in the short term, to reducing exudate, alleviating malodor, and improving upper limb lymphedema associated with advanced breast cancer wounds. It also appears to synergistically promote clinical improvement in laboratory parameters—including markers of infection, anemia, and hypoalbuminemia—as well as in pleural effusion. However, HBOT did not reduce wound size, nor did it prevent the subsequent progression of the disease, which ultimately led to the patient's death due to infection. Therefore, the primary value of HBOT in this case lies in short‐term symptom control and quality of life improvement, rather than in achieving structural healing or long‐term survival benefits. In clinical practice, for patients with similar advanced cancerous wounds, HBOT may be considered as an adjunct within comprehensive palliative care, to be used judiciously alongside infection control, nutritional support, lymphedema management, and wound care. However, it should not be overestimated as a means to reverse tumor progression or as a substitute for curative treatment. Its precise indications and efficacy warrant further validation through prospective controlled studies.

## Author Contributions

**Fengrong Tang:** conceptualization, investigation, funding acquisition, writing – original draft, writing – review and editing, validation, project administration, resources, supervision. **Xiaoxiao Duan:** investigation, validation, formal analysis. **Lei Zhang:** conceptualization, investigation, writing – review and editing, resources, project administration. **Xiaochen Mo:** investigation, validation, formal analysis. **Shufang Lu:** investigation, validation, formal analysis, data curation.

## Funding

The authors have nothing to report.

## Ethics Statement

We have clarified in the manuscript that the study was approved by Minhang Branch, Fudan University Shanghai Cancer Center (ethical approval number: 2024 Research No. 011).

## Consent

Informed consent was obtained from the patient and their families for the publication of the case details.

## Conflicts of Interest

The authors declare no conflicts of interest.

## Data Availability

Data sharing not applicable to this article as no datasets were generated or analyzed during the current study.
